# Impaired Proteostasis is Linked to Neurological Pathology in a Zebrafish NGLY1 Deficiency Model

**DOI:** 10.1002/jimd.70050

**Published:** 2025-06-05

**Authors:** Aviv Mesika, Golan Nadav, Sapir Ben‐David, Limor Kalfon, Chen Shochat, Rana Nasra, Alejandro Livoff, David Karasik, Tzipora C. Falik‐Zaccai

**Affiliations:** ^1^ Institute of Human Genetics, Galilee Medical Center Nahariya Israel; ^2^ Azrieli Faculty of Medicine Bar Ilan University Safed Israel; ^3^ Department of Pathology Galilee Medical Center Nahariya Israel

**Keywords:** amyloids, aquaporin 1, NGLY1 deficiency, proteostasis, zebrafish

## Abstract

NGLY1 is a key enzyme in the process of misfolded protein deglycosylation. Bi‐allelic pathogenic variants in *NGLY1* cause N‐glycanase deficiency, also known as congenital disorder of deglycosylation (NGLY1‐CDDG). This rare and multisystem autosomal recessive disorder is linked to a variable phenotype of global developmental delay, neuromuscular abnormalities, and alacrima, and it lacks effective treatment. We have studied the possible underlying mechanisms for the neuromuscular and ophthalmic phenotypes in an *ngly1‐*deficient zebrafish model carrying a similar genetic variant that has also been identified in previously reported patients. We investigated phenotypic, biochemical, and molecular details underlying ngly1 deficiency using a zebrafish model. *ngly1‐*deficient zebrafish phenotypes were characterized using histological staining, transmission electron microscopy (TEM), and micro‐CT imaging. Furthermore, fish brain molecular and biochemical characterization was performed by gene expression analysis and immunoblotting techniques. Impaired proteostasis was evident in the brain of the mutant zebrafish, including accumulation of poly‐ubiquitinated proteins and amyloid fibril aggregation. The mutant fish featured neuromuscular abnormalities and significant aquaporin1‐protein reduction in the eyes and brain. The zebrafish model of NGLY1 deficiency provides an ideal platform for studying the molecular and biochemical mechanisms underlying NGLY1‐CDDG in humans. Our novel findings of impaired protein homeostasis encompassing amyloid fibril aggregation (folding) and poly‐ubiquitinated protein accumulation (degradation) in the brains of mutant zebrafish offer new insights into the brain pathology associated with NGLY1 deficiency. These discoveries may also advance our understanding of other neurodegenerative disorders and facilitate the identification of potential therapeutic targets.

## 1 Introduction

Bi‐allelic pathogenic genetic variants in *NGLY1* cause N‐glycanase deficiency (OMIM **#** 615273), a condition belonging to the congenital disorder of deglycosylation CDDG cluster of disorders, and also known as NGLY1‐CDDG. N‐glycanase deficiency is a rare autosomal recessive disorder [1, 2], with variable multi‐organ phenotypes [3, 4]. Since the first report of a patient with NGLY1 deficiency in 2012 [1], approximately 100 cases have been reported worldwide [4], and it is possible that many more undiagnosed patients are suffering from this devastating disease with no effective treatment [5, 6]. Moreover, the natural history of the disease remains unclear, since this severe disorder is lethal and many patients die at an early age [7]. We have identified four patients with NGLY1‐CDDG due to a nonsense genetic variant in exon 9 of *NGLY1* [8]. In general, the nervous system pathology of the patients manifested by severe global developmental delay, movement disorders and seizures [4]. Morphological changes in the musculoskeletal (MSK) system such as scoliosis, muscle atrophy, and osteopenia are clinical manifestations correlating to the nervous system abnormality [7]. Furthermore, NGLY1‐deficient patients demonstrate a hallmark phenotype of congenital alacrima (lack of tears that range from absence to hyposecretion in some patients), which is observed in several rare disorders [9]. Early‐onset liver disease and elevated liver transaminases might also be present in NGLY1‐CDDG disorder, but evidence suggests that, in some cases, patients recover from the hepatic disease and subsequently show normal liver functions [8]. N‐glycanase, encoded by NGLY1, is an enzyme with a crucial role in the endoplasmic reticulum‐associated protein degradation (ERAD) pathway [10]. Its multifunctional roles include deglycosylation of misfolded glycoproteins during the ubiquitin‐mediated protein degradation process [11, 12, 13], mediation of signaling pathways [14, 15, 16, 17] and involvement in regulation of mitochondrial physiology [18]. A recent publication demonstrated that NGLY1 deficiency affected water channel (aquaporin) expression, which may underlie the phenotype of alacrima [19]. Several animal models have been established to explore the mechanisms behind the phenotype of NGLY1 deficiency, including mouse [20, 21], rat [22] and Drosophila [15]. However, the typical early mortality of NGLY1‐deficient animals makes it difficult to analyze the gene's function in adults and has provided only a partial understanding of the course of disease progression [23].

We have previously created a homozygous *ngly1* ^*(−/−)*^ zebrafish bearing a nonsense genetic variant in exon 9 similar to the genetic variant in our patients [24]. This loss‐of‐function zebrafish model exhibits abnormalities in both the nervous and musculoskeletal systems. The zebrafish model allows for the study of late‐onset manifestations of NGLY1 deficiency as this model progresses from juvenile to mature stages. Indeed, this study documents a relatively slow progression of the morphological phenotypes of the mutated fish, along with significant reduction of aquaporin‐1 protein in the eyes and brain, and impaired protein homeostasis in the brain. This impairment encompasses disruption of the ubiquitin pathway and amyloid aggregation. Zebrafish are excellent models for studying rare and neurodegenerative human diseases [25]. The zebrafish model might provide a comprehensive understanding of the role of NGLY1 in the development and survival of the nervous system [25, 26] and may serve as a platform for studying possible innovative therapies for NGLY1‐CDDG [27, 28].

## 2 Materials and Methods

### 2.1 Zebrafish Maintenance

All animal research followed a protocol approved by BIU IACUC (# 023_b15280_80). Zebrafish ( *Danio rerio* ) of AB strain were maintained at 28°C, under 14 h light:10 h dark cycles. Before the experiments, adult and larval fish were euthanized by immersion in tricaine methane sulfonate (MS222) 0.4% and subsequently placed on ice.

The zebrafish model is described [24]. In brief, germline transmission of a mutation in F1 progeny was verified by crossing the adult founders (F0) to WT fish. F1 fish (heterozygous for the frameshift mutation) were then crossed to WT to obtain F2, which, by inbreeding, produced homozygosity to a deletion of 19 bp (c.1759–1777) resulting in p.met517fs (*ngly1* ^*(−/−)*^). This mutation causes the appearance of a premature stop codon creating a 535 amino acid protein instead of the 644 amino acid full length of ngly1 zebrafish protein [24]. To minimize an off‐target effect, adult fish carrying the 19 bp deletion (F2) were further outcrossed to WT; here we present results for generations F3 and later. In the experiments, *ngly1* ^*(−/−)*^ mutant fish are compared to WT *ngly1* ^*(+/+)*^ control siblings.

### 2.2 Protein Extraction and Western Blotting (WB)

Proteins were extracted from eyes and brain (1Y) of post‐mortem wild type (WT) and mutant fish. The immunoblotting analysis was performed with anti‐aqp1a.1 antibody kindly provided by Dr. Gordon Cramb (University of St Andrews, Scotland) as well as anti‐UBB antibody (Santa Cruz Biotechnology, Dallas, TX, USA; Cat. No. sc‐166 553). Finally, the membrane was analyzed using G:BOX (Syngene, Frederick, WA, USA) with a chemiluminescence light.

### 2.3 Histological Staining

Brain, liver, and muscle biopsies from WT and mutant fish were embedded in paraffin according to standard protocols [29]. Sections were dehydrated three times in xylene for 2 min, then dehydrated in 100% alcohol and washed with 80% alcohol and tap water. Then, the slides were stained with hematoxylin and eosin or Congo Red stain using a commercial kit (AMY‐2 by Scytek, Logan, UT, USA) according to the manufacturer's instructions. Myofibers cross‐section area (CSA) was measured using Image J software (National Institutes of Health, Bethesda, MD, USA). All of the quantifications were done by three observers blinded to fish genotype.

### 2.4 Immunohistochemistry

Brains from adult fish were fixed in a 4% paraformaldehyde solution and serially cut to 4 μm sections [30]. All sections were incubated overnight at 4°C with anti‐UBB (1:200, Santa Cruz Biotechnology, Dallas, TX, USA; Cat. No. sc‐166 553) and anti‐amyloid fibril (1:200, Invitrogen, Beverly, MA, USA; Cat. No. PA5‐77843) antibodies. Specimens were imaged with a Nikon Eclipse Ti and a Nikon C2 confocal scanner.

### 2.5 Skull Morphology

Bones of 1‐year‐old *ngly1* ^(−/−)^ and *ngly1* ^(+/+)^ fish were imaged with a SkyScan 1172 μCT scanner (Bruker, Kontich, Belgium) at 12 μm isotropic voxel size, according to the manufacturer's instructions. Scans were taken over 360°, with a 0.40° rotation step. A region of interest traced around the whole zebrafish was thresholded for hydroxyapatite using a manually determined global threshold. The dentary‐basihyal angle was measured as previously described [31].

### 2.6 Alcian Blue and Alizarin Red Double Staining

Zebrafish larvae at the age of 7 days' post‐fertilization (*dpf*) were stained with Alcian blue/Alizarin red double staining for skeletal examination as previously described [31]. The larvae were photographed using a Leica microscope camera DFC365 FX (Leica Microsystems, Wetzlar, Germany), focusing on the cranial region. In cranial region, percentage of proximal notochord's calcification was quantified using ImageJ software (National Institutes of Health, Bethesda, MD, USA), and compared between *ngly1* ^*(−/−)*^ and *ngly1* ^*(+/+)*^ larvae.

### 2.7 Transmission Electron Microscopy (TEM)

Brains from adult fish were fixed in 2.5% glutaraldehyde. TEM imaging was performed as previously described [32], with a focus on the periventricular gray zone (PGZ) and the lateral division of valvula cerebelli (Val) areas due to locally observed abnormalities in histological staining. We performed an analysis of mitochondrial morphology using the Form Factor metric [33], calculated by the formula:

Form Factor = (4 × π × mitochondrial area)/(mitochondrial perimeter) [2]. Higher values of this parameter indicate a more circular and regular shape, while lower values reflect an elongated or irregular morphology of mitochondria. The quantification was carried out in a blinded fashion.

### 2.8 Zebrafish Serum Biochemistry Measurements

Serum samples were collected from larval and adult zebrafish as previously described [34]. Serum ALT and AST activities were measured using commercial diagnostic kits, according to the manufacturer's instructions (Abcam, Cambridge, UK).

### 2.9 RNA Sequencing

RNA‐seq analysis was performed on total RNA extracted from the brains of adult *ngly1* ^*(−/−)*^ and *ngly1* ^*(+/+)*^ zebrafish siblings (*n* = 12, each group). RNA‐seq libraries were prepared using the NEBNext RNA Library Prep kit (New England BioLabs Inc.). The indexed libraries were pooled and sequenced (75bpsX1) on NextSeq (Illumina). Raw reads were aligned to the zebrafish genome (GRCz11) with TopHat (a splice junction mapper) on the PARTEK platform.

### 2.10 mRNA Level Evaluation

cDNA amplicons for *aqp1a.1, ube3a*, and *ubb* genes were evaluated using RT‐PCR, with *gapdh* used as a reference gene and normalized to normal controls. Delta–delta cycle threshold (CT) was calculated as previously described [31].

### 2.11 Statistical Analysis

All statistical analyses were performed using T‐tests in Prism software (GraphPad Software, Boston, MA, USA).

For the RNA‐seq dataset, gene lists were filtered to include only genes that passed a *p*‐value threshold of 0.001.

## 3 Results

### 3.1 NGLY1‐Deficient Zebrafish Model Expresses the Hallmarks of NGLY1‐CDDG

#### 3.1.1 Zebrafish Brain Morphology Confirms Massive Neural Cell Loss

**FIGURE 1:**
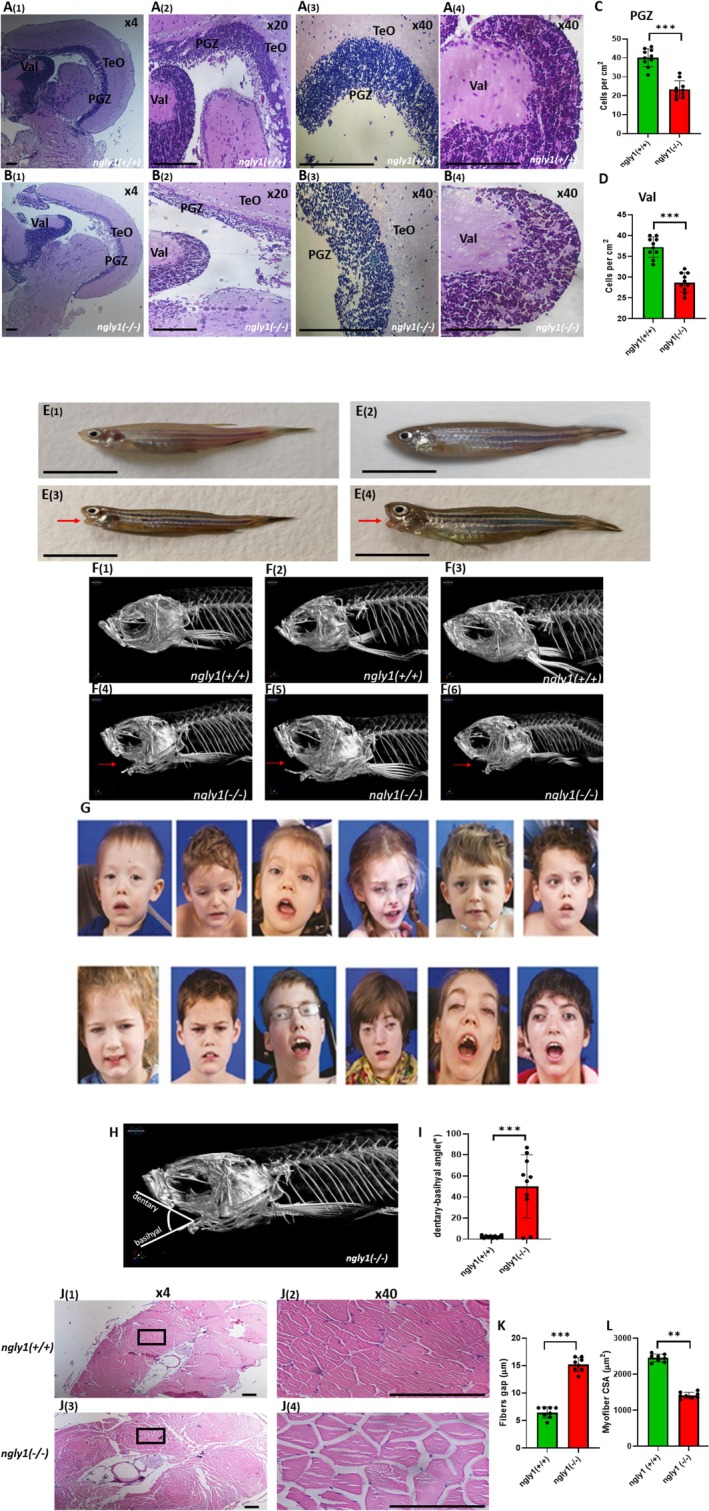
**Adult *ngly1*** ^***(−/−)***^ **zebrafish display the hallmarks of NGLY1‐CDDG**. Representative images of *ngly1* ^*(+/+)*^ (**A**_**(1–4)**_) and *ngly1* ^*(−/−)*^ (**B**_**1–4**_) coronal brain sections (slides, hematoxylin and eosin staining) displaying massive loss of neural cells in *ngly1* ^*(−/−)*^. Magnifications: A_1_, B_1_‐x4, A_2_, B_2_‐x20 and A _[_ 3, 4_]_, B _[_ 3, 4_]_‐x40. **(C, D)** Quantification of cell numbers in the periventricular gray zone (PGZ) **(C)** and lateral division of valvula cerebelli (Val) **(D)**. ***T‐test *p* < 0.001 (*N* = 10 in each group). Scale bar: 100 μm. Gross morphology of adult **(E**_(**1**)_–**E**_(**2**)_) *ngly1* ^*(+/+)*^ and (**E**_(**3**)_–**E**_(**4**)_) *ngly1* ^*(−/−)*^ fish. μCT imaging of *ngly1* ^*(+/+)*^ (**F**_(**1**)_–**F**_(**3**)_) and *ngly1* ^*(−/−)*^ (**F**_(**4**)_–**F**_(**6**)_); red arrows represent the basihyal bone dislocation. **(G)** Facial features of human patients include upturned nasal tip, hypotonic facies, ptosis, brachycephaly, thinned facies, hollowed cheeks, and visible zygomatic arches. ** **(H)** Schematic representation of dentary‐basihyal angle which was significantly greater in *ngly1* ^(−/−)^. **(I)** Quantification of dentary‐basihyal angle. ***T‐test, *p* < 0.001 (*N* = 10 in each group). Scale bar: 1 cm. **(J)** Coronal trunk section histology staining (hematoxylin and eosin) of (J_(1)_–J _[_ 2_]_) *ngly1* ^*(+/+)*^ and (J_(3)_–J _[_ 4_]_) *ngly1* ^*(−/−)*^. *ngly1* ^*(−/−)*^ showed reduced in muscle fiber content of trunk skeletal muscle. Zoom‐in of muscle regions in (J _[_ 1_]_), (J _[_ 3_]_)‐X4 and (J _[_ 2_]_), (J _[_ 4_]_) –X40. **(K)** Quantification of gaps between muscle fibers, *** T‐test *p* < 0.001 (*N* = 8 in each group). **(L)** Quantification of muscle fibers CSA, ** T‐test *p* < 0.01 (*N* = 8 in each group). Scale bar:100 μm. **Reprinted from Genetics in Medicine. Prospective phenotyping of NGLY1‐CDDG, the first congenital disorder of deglycosylation. Lam, C., Ferreira, C., Krasnewich, D., Toro, C., Latham, L., Zein, W. M., Lehky, T., Brewer, C., Baker, E. H., Thurm, A., Farmer, C. A., Rosenzweig, S. D., Lyons, J. J., Schreiber, J. M., Gropman, A., Lingala, S., Ghany, M. G., Solomon, B., Macnamara, E., Davids, M., Wolfe, L. 19, 160–168 (2017). with permission from Elsevier**.

Histological examination of coronal sections of adult *ngly1* ^*(−/−)*^ zebrafish identified massive neural cell loss in the periventricular gray zone (PGZ) [*p* < 0.001] and in the lateral division of valvula cerebelli (Val) [*p* < 0.001], which was not apparent in their *ngly1* ^*(+/+)*^ siblings (Figure 1A–D).

#### 3.1.2 Adult *ngly1* ^*(−/−)*^ Zebrafish Feature a Shorter Snout, Protruding Basihyal Bones, and Reduction in Muscle Microstructural Integrity

Micro‐CT (μCT) imaging of the entire adult fish was conducted to characterize the anatomical phenotype of the adult skeleton. The analysis found a significantly increased [*p* < 0.001] dentary‐basihyal angle in *ngly1* ^(−/−)^ as compared to *ngly1* ^(+/+)^ zebrafish (Figure 1E–I). This phenotype resembles the progressing human facial phenotype with mandible drop at a later age. Moreover, histological staining confirms that the muscle structure of NGLY1‐deficient (*ngly1* ^*(−/−)*^) fish exhibits larger gaps between muscle fibers [*p* < 0.001] and a reduction in myofiber CSA [*p* < 0.001], indicating reduced muscle fiber content compared to the control group (Figure 1J–L).

#### 3.1.3 Adult *ngly1* ^*(−/−)*^ Zebrafish Show Significantly Reduced Aquaporin 1 (aqp1a.1) Levels

**FIGURE 2:**
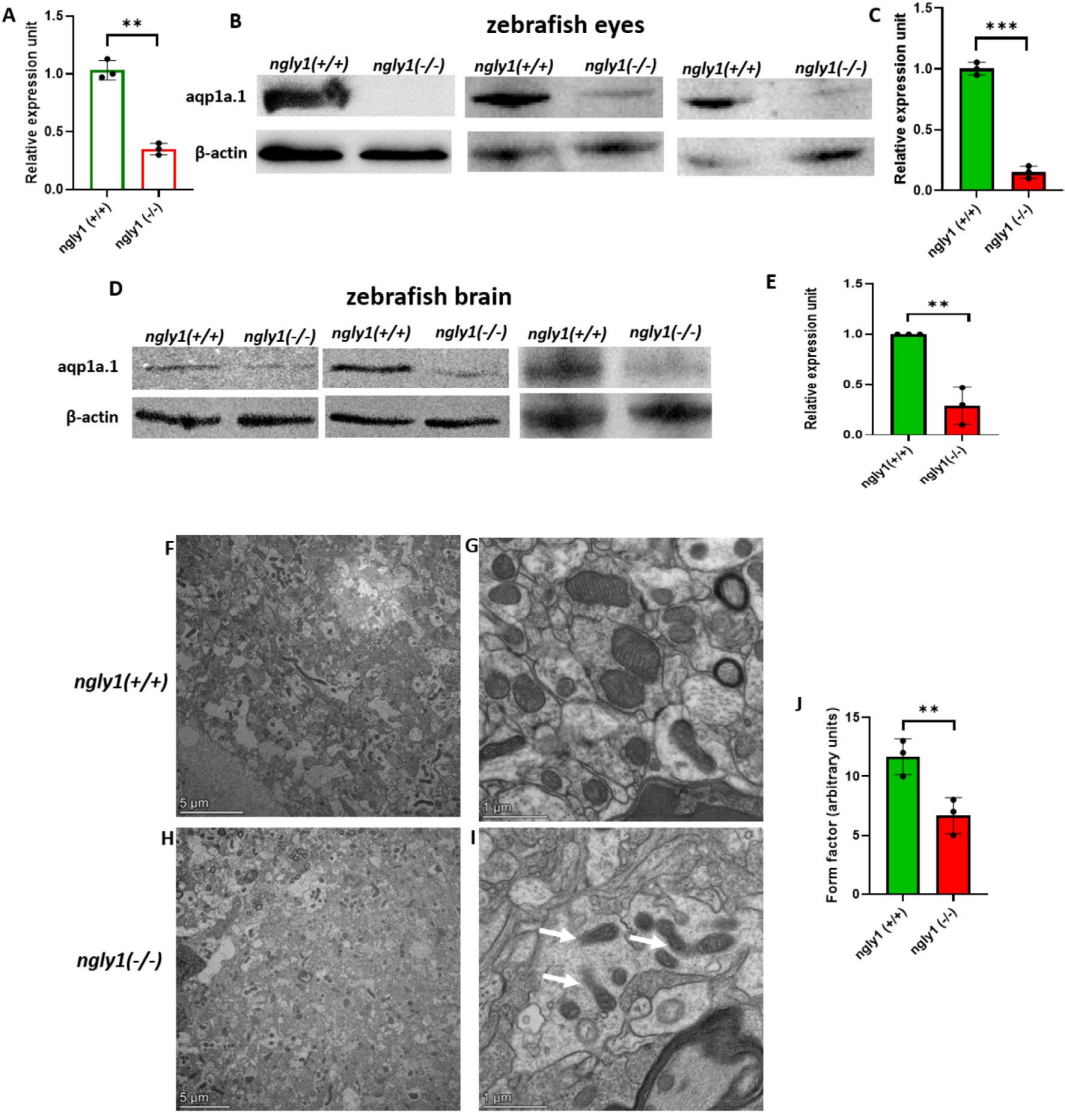
**Adult *ngly1*** ^***(−/−)***^ **fish feature cellular effects resulting from NGLY1 deficiency**. **(A)** RT‐qPCR quantification of *aqp1a.1* mRNA levels in zebrafish eye. ** T‐test *p* < 0.01 (*ngly1* ^*(+/+)*^ *n* = 3, *ngly1* ^*(−/−)*^ *n* = 3). (**B**–**E**) Western blot analysis of aqp1a.1 protein from adult zebrafish eyes (**B**, **C**); *** T‐test *p* < 0.001 (*ngly1* ^*(+/+)*^ *n* = 3, *ngly1* ^*(−/−)*^ *n* = 3), and brain **(D, E)** ** T‐test *p* < 0.01 (*ngly1* ^*(+/+)*^ *n* = 3, *ngly1* ^*(−/−)*^ *n* = 3). β‐actin was used as a loading control. (**F**–**I**) Transmission electron microscopy images showing mitochondrial fragmentation in the mutant zebrafish brain (white arrows). **(J)** Fragmented mitochondria were quantified by Form Factor metric, calculated by the formula: Form Factor = (4 × π × mitochondrial area)/(mitochondrial perimeter) [2]. ** T‐test *p* < 0.01, (*N* = 3 in each group).

Adult *ngly1* ^*(−/−)*^ zebrafish exhibited significantly reduced levels of *aqp1a.1* mRNA expression in the eyes (*p* < 0.01). Correspondingly, aquaporin 1 (Aqp1a.1) protein levels were also significantly decreased in both the eye (*p* < 0.001) and brain (*p* < 0.01) of mutant fish compared to *ngly1* ^*(+/+)*^ controls (Figure 2A–E).

### 3.2 Mitochondrial Fragmentation in the Brain of Adult *ngly1* ^*(−/−)*^ Zebrafish

Next, cellular‐level brain injury in adult *ngly1* ^*(−/−)*^ zebrafish was explored by TEM. The analysis showed a significantly higher percentage of mitochondria with fragmented membrane morphology [*p* < 0.01] in the brain of the mutant fish vs. their *ngly1* ^*(+/+)*^ siblings (Figure 2F–J).

### 3.3 Liver Morphology and Functions Are Normal in the Mutant Zebrafish

To determine whether *ngly1* ^*(−/−)*^ induces liver damage, the liver functions of larval and adult fish were examined by measurement of alanine aminotransferase (ALT) and aspartate aminotransferase (AST) activity. Both larval and adult *ngly1* ^*(−/−)*^ fish showed similar levels of ALT/AST activity compared to WT fish. Both mutant and WT fish had normal liver morphology (Figure S1 A–D).

### 3.4 *ngly1* Is Not Involved in Early Bone Morphogenesis in Zebrafish

In order to study musculoskeletal system development, whole mount skeletal staining by Alizarin red/Alcian blue for bone and cartilage was performed. The effects of *ngly1* loss‐of‐function on bone/cartilage development were analyzed in 7 *dpf ngly1* ^*(−/−)*^ and WT siblings. Bone morphological alterations were not identified in *ngly1* ^*(−/−)*^ fish compared to controls (Figure S2 A–D).

### 3.5 Impaired Proteostasis in the Brain of the NGLY1‐Deficient Zebrafish Model

#### 3.5.1 Downregulation of Ubiquitin‐Related Genes and Amyloid Aggregation in the Brains of NGLY1‐Deficient Zebrafish

**FIGURE 3:**
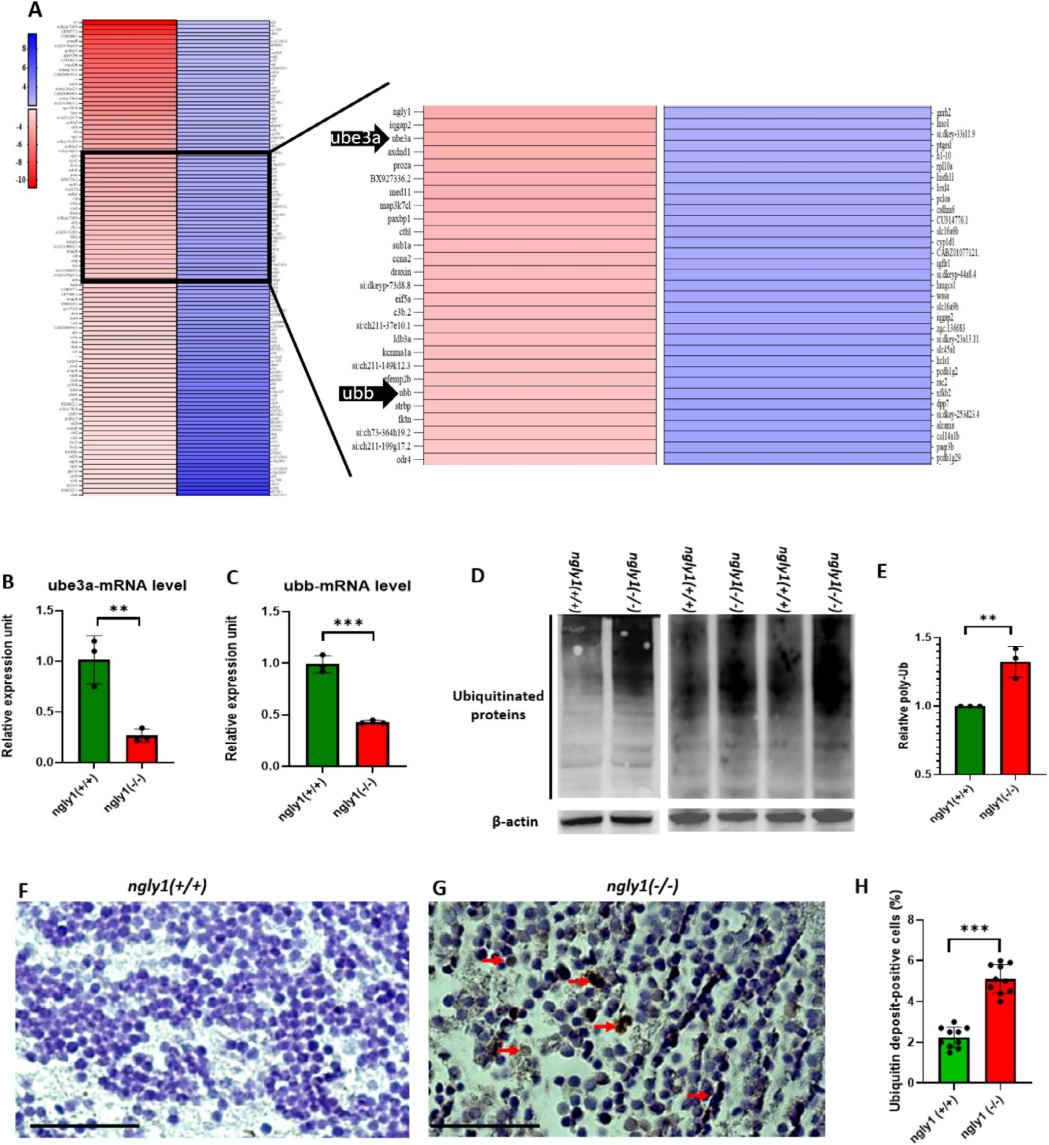
**Impaired ubiquitin‐mediated protein degradation in the adult *ngly1*** ^***(−/−)***^ **fish brain. (A)** Heat map presentation of RNA sequencing of zebrafish brains, red = downregulation and blue = upregulation, the scale indicates fold change values. **(B, C)** RT‐qPCR quantification of *ube3a* **(B)** and *ubb* **(C)** mRNA levels. ** T‐test *p* < 0.01, *** T‐test *p* < 0.001 (*ngly1* ^*(+/+)*^ *n* = 3, *ngly1* ^*(−/−)*^ *n* = 3, *ube3a*, *ubb*). **(D)** Western blot analysis of zebrafish brain with an antibody against poly‐ubb; β‐Actin was used as a loading control. **(E)** Quantification of western blot‐determined ubiquitinated protein levels. **T‐test *p* < 0.01 (*ngly1* ^*(+/+)*^ *n* = 9, *ngly1* ^*(−/−)*^ *n* = 9). Representative immunohistochemistry images of *ngly1* ^*(+/+)*^ **(F)** and *ngly1* ^*(−/−)*^ **(G)** zebrafish brains periventricular gray zone (PGZ) reacted with anti‐poly‐ubb antibody, **(H)** Percentage of ubiquitin deposit‐positive cells. ***T‐test *p* < 0.001 (*N* = 10 in each group). Red arrows indicate cells with ubiquitinated protein accumulation.

**FIGURE 4:**
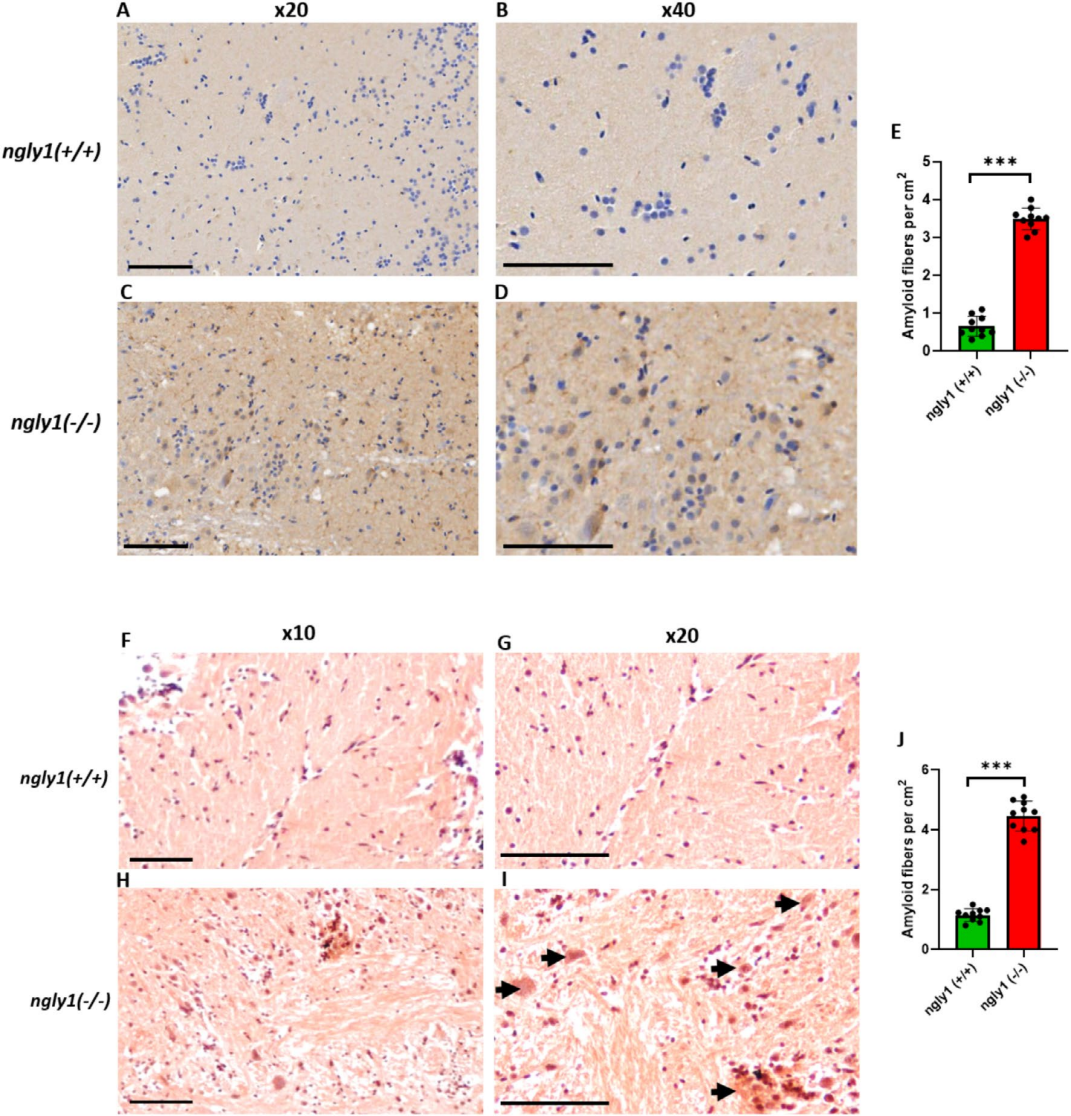
**Amyloid aggregation in adult NGLY1‐deficient zebrafish brains**. Representative histological images of *ngly1* ^*(+/+)*^ **(A, B, F, G)** and *ngly1* ^*(−/−)*^ **(C, D, H, I)** zebrafish brain and optic tectum (TeO) reacted with an antibody against amyloid fibrils (**A**–**D**) and Congo Red (**F**–**I**), demonstrating significant aggregation of amyloid fibrils (black arrows) at the intracellular space of the mutant fish brain. Magnifications: (F, H): X10, (A, C, G, H): X20, and (B, D): X40. Scale bar: 100 μm. **(E, J)** Quantification of amyloid fibers by Immunohistochemistry and Congo Red staining, respectively. ***T‐test, *p* < 0.001 (*N* = 10 per group).

To investigate the impact of the mutant ngly1 on the ERAD pathway and protein degradation process, brain pathology was analyzed in adult fish. Bulk RNA‐seq in adult zebrafish brains identifies downregulation of genes related to the ubiquitin‐mediated protein degradation process that was further confirmed by RT‐qPCR (Figure 3A). Specifically, there was a significant reduction in *ubb* (ubiquitin) [*p* < 0.001] and *ube3a* (ubiquitin E3 ligase) [*p* < 0.01] mRNA levels in the mutant vs. normal fish (Figure 3B,C). Finally, a significant accumulation of poly‐ubiquitination proteins [*p* < 0.01] was measured in the *ngly1* ^*(−/−)*^ adult zebrafish brain (PGZ area) using WB and immunohistochemistry analysis (Figure 3D–H). Immunohistochemical staining and Congo Red dye analysis revealed a significantly generalized aggregation [*p* < 0.001] of amyloid fibrils in the intracellular space of the brain in mutant fish (Figure 4A–J) vs. controls.

## 4 Discussion

The present study in an adult NGLY1‐deficient zebrafish model yielded novel findings regarding the brain pathology and molecular characteristics of NGLY1‐CDDG. The model was based on a homozygous pathogenic genetic variant identified in four patients from two related Druze families who presented with clinical features of NGLY1‐CDDG [8]. The nonsense genetic variant was created in zebrafish exon 9, located similarly to the genetic variant in the patients [24]. This study revealed impaired proteostasis, including downregulation of ubiquitin‐mediated protein‐degradation genes and amyloid aggregation in the fish brain, which may be related to the neurological phenotype in humans. Attempts to create animal models that mimic NGLY1‐CDDG have been made in mice [20, 21], rats [22], and drosophila [15]. However, the semi‐lethal nature of Ngly1‐deficient animals made it difficult to analyze its function in adults and provided only a partial understanding of the pathophysiology underlying disease progression [23]. We hypothesize that our zebrafish model offers an advantage in terms of survival into adulthood. This extended survival in the *ngly1* ^*(−/−)*^ zebrafish may be partly due to the presence of residual NGLY1 protein [24], which could lessen the severity of a complete loss‐of‐function phenotype. Another possible explanation is a compensatory mechanism described in zebrafish, in which nonsense‐mediated mRNA decay (NMD) can trigger transcriptional adaptation to counteract the effects of premature stop codons [35]. Such a mechanism may operate in our model, supporting survival into adulthood despite the introduction of a premature stop codon. The mutated fish model presented here offers unique opportunities for modeling NGLY1‐CDDG cluster diseases by morphological examination, dynamic behavioral testing, and biochemical analyses of the whole organism at both larvae and adult stages.

The mutant zebrafish featured a distinct brain morphology, with loss of neural cells in the PGZ and Val, two areas in the brain that are responsible for motor and sensory functions [36, 37]. This study demonstrates, for the first time, that the NGLY1 pathogenic variant induces morphological fragmentation of brain mitochondria in the PGZ and Val regions (Figure 2F–J). Furthermore, due to the inherent limitations of TEM, our findings cannot conclusively establish that mitochondrial damage is confined solely to neurons. Nevertheless, we were able to localize the mitochondrial impairment to the same brain region, the PGZ and Val, where neural loss is observed. These findings align with the work of Kong et al. [18], who demonstrated that N‐glycanase deficiency disrupts mitochondrial function, leading to a significant reduction in mitochondrial membrane potential and an elevated oxidant burden within the mitochondrial matrix. A plausible explanation for this phenomenon lies in the mitochondria's response to cellular stress, such as the loss of membrane potential, which triggers a shift toward mitochondrial fission, resulting in fragmented mitochondria [38]. This adaptive response further underscores the intricate interplay between proteostasis and mitochondrial dynamics in the context of NGLY1 deficiency. These findings support a causative correlation between a pathogenic (nonsense) genetic variant of *NGLY1*, fragmented mitochondria, and the neurological phenotype in zebrafish and possibly in humans. Notably, NGLY1 plays several crucial roles, including the deglycosylation of misfolded glycoproteins in ubiquitin‐mediated protein degradation [11], modulation of key signaling pathways [14], and regulation of mitochondrial function [18]. It is highly plausible that the brain pathology associated with NGLY1 deficiency results from the interplay of these mechanisms.

NGLY1 deficiency is defined as a neuromuscular disorder [39], with patients displaying MSK structural abnormalities that are defined as secondary phenotypes of the disease [1]. Adult (1‐year‐old) mutant fish exhibited significant muscular atrophy previously reported in human patients [7] with no significant differences in size and weight between the mutant fish and the control group. In addition, a unique phenomenon of *mandibular* and branchial‐arch dislocation was observed in the mutant compared to the WT fish. Specifically, the basihyal bone protruded and was dislocated (Figure 1E–I) and was almost identical to the phenomena of open‐tented mouth in NGLY1‐CDDG patients [2]. To further investigate the MSK system, we performed Alcian blue combined with Alizarin red staining to visualize cartilage and bone, respectively, at the larval stage (7 *dpf*). This method enabled us to assess the impact of ngly1 loss‐of‐function on skeletal development. Analysis of notochord ossification in *ngly1* ^*(−/−)*^ and *ngly1* ^*(+/+)*^ siblings revealed no significant differences in bone formation (Figure S2). These findings indicate that the zebrafish model of NGLY1 deficiency reflects the progressive trajectory of the disease, showing no skeletal abnormalities at the larval stage, while adult fish exhibit marked skeletal deformities likely secondary to chronic muscle weakness. It is therefore hypothesized that the trunk skeletal muscle atrophy observed in ngly1‐deficient fish arises from peripheral neuropathy [24], potentially underlying the neuromuscular phenotype and mirroring key features of the human disorder.

The third system involved in NGLY1‐CDDG is the eyes. Alacrima is a rare clinical symptom that manifests in several genetic disorders [9]. Tambe et al. demonstrated the impact of NGLY1 deficiency on the regulation of the water‐channel (aquaporin) family and suggested it as a potential mechanism of alacrima [19]. The present work reports for the first time an eye phenotype in a ngly1‐deficient animal model where the eyes of the mutant fish showed a similar pattern of aquaporin1 level as described in the human patients' fibroblasts [19]. These findings are consistent with previous reports indicating that N‐Glycanase 1 regulates aquaporin expression at the transcriptional level and suggest a similar mechanism may be present in human cells [19]. The results further support a possible causative link between AQP1 expression level and congenital alacrima in NGLY1‐deficient patients [19]. Since no other significant ocular pathology has been reported in NGLY1‐deficient patients, this work focused on the expression pattern of AQP1 in the eyes and the alacrima phenomenon [40]. Of special note is the work of Preston and colleagues from the early 1990s, which did not describe any eye phenotype in patients lacking AQP1 [41]. This suggests that the lack of AQP1 is not the exclusive cause of alacrima, but rather, plays a role in a complex redundant mechanism underlying normal tear production and secretion in humans. It is possible that the complex alacrima phenotype in a vast majority of NGLY1‐CDDG patients worldwide is caused by the combined deficiency of AQP1 and NGLY1 [4].

Finally, accumulation of poly‐ubiquitinated proteins is a possible biomarker related to the causative mechanism of NGLY1‐CDDG [22]. The current analysis demonstrated impaired ubiquitin‐mediated protein degradation which led to accumulation of poly‐ubiquitinated proteins in the *ngly1* ^*(−/−)*^ adult zebrafish brain, similar to the reported findings in a rat model [22]. Moreover, the mutation in *ngly1* led to downregulation of ubiquitination gene expression, aligning with the significant reduction of expression in two important genes of the ubiquitination pathway—*ube3a* (ubiquitin E3 ligase) and *ubb* (ubiquitin B) in the mutant compared to WT adult fish. Studies have previously shown that *UBE3A* loss of function leads to Angelman syndrome, a complex severe neurological genetic disorder, suggesting that *UBE3A* has a critical role in the normal development and function of the CNS [42]. Additionally, *UBB* dysregulation led to a progressive degenerative disorder affecting neurons [43]. The mechanism underlying *ube3a* downregulation in the mutant zebrafish brain remains unclear. However, previous studies have reported decreased UBE3A expression in Alzheimer's disease [44], although it is still uncertain whether this reduction is a cause or a consequence of amyloid accumulation. This documented association between reduced UBE3A levels and neurodegenerative pathology highlights its potential relevance to the underlying mechanism of NGLY1‐CDDG.

The downregulation of genes involved in the ubiquitination process may contribute to the neurological phenotypes observed in *ngly1* ^*(−/−)*^ zebrafish. As such, targeting components of the ubiquitination pathway could represent a novel therapeutic strategy. One potential target is USP14, a deubiquitinating enzyme associated with the proteasome that edits polyubiquitin chains and can delay substrate degradation. In NGLY1 deficiency, inhibiting USP14 may enhance proteasomal activity by facilitating the more efficient degradation of polyubiquitinated substrates [45]. This approach could be further potentiated by combining USP14 inhibition with other proteostasis‐boosting strategies, including autophagy inducers (mTOR inhibitors) to help restore protein homeostasis and promote cellular function [46].

Another original aspect of the impaired protein degradation pathway reported here was the generalized amyloid aggregation in the mutant adult fish brain. This finding contradicts a recent study that described amorphous protein aggregation without any significant amyloid component in a particular type of cortical neurons from iPSCs [47] derived from NGLY1‐deficient patients with five different pathogenic genetic variants. The presence of generalized amyloid aggregation observed in our study can be explained by the fact that the zebrafish model mimicked the complexity of the whole brain as a complex organ and expressed the effects of other types of cells that are of great significance in the CNS. The significant reduction in aqp1a.1 protein levels in the mutant vs. WT fish brain provides an additional explanation for the amyloid aggregation phenomenon as AQP1 has been shown to inhibit amyloid genesis in the nervous system [48]. Since aggregation of amyloids is a mechanism underlying several neurodegenerative diseases [49], repurposing drugs already approved to treat other neurodegenerative diseases may be cost effective and applicable for the treatment of NGLY1‐deficient patients.

Several patients with NGLY1‐CDDG were reported to present elevated liver transaminase levels and liver fibrosis [4]. The mutant fish studied here showed normal liver morphology and function, as measured by histological staining and ALT and AST activity (Figure S1). The results of this study suggest that the liver might have mechanisms to adjust and recuperate from the cellular pathology occurring when *NGLY1* is disrupted. This aligns with the clinical observation that some of the patients recovering from hepatic disease regain normal liver function [8]. However, further characterization of the liver at the cellular level is essential and might explore additional molecular pathways involved in NGLY1‐CDDG liver disease.

**FIGURE 5:**
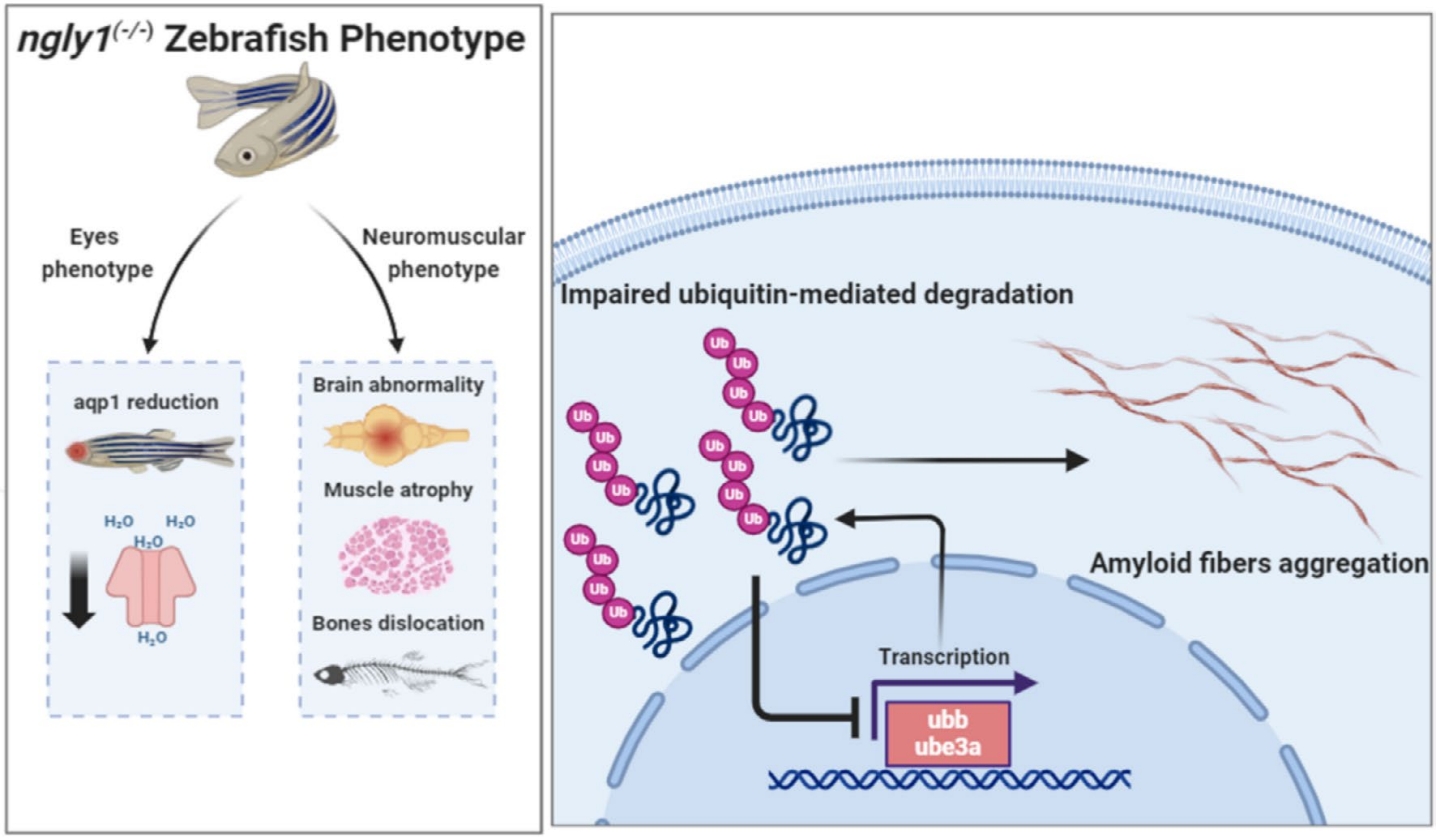
**Schematic representation of *ngly1*** ^***(−/−)***^ **zebrafish phenotypes and brain abnormality**. The NGLY1‐deficient zebrafish model features key NGLY1‐CDDG clinical hallmarks. Impaired ubiquitination and amyloid fibrils aggregation are involved in *ngly1* ^*(−/−)*^ brain pathology.

In summary, this work documents the natural history and the progression of NGLY1 deficiency in larval and adult stages of a zebrafish model carrying a human genetic variant. The adult mutant fish featured a neuromuscular phenotype reflected by loss of neural cells and mitochondrial fragmentation in the brain. These nervous system abnormalities may have led to the secondary MSK phenotype of reduced muscle mass and branchial apparatus bone dislocation. Moreover, biochemical analyses showed a significant reduction in aquaporin 1 protein level in the eyes and brain of adult *ngly1* ^*(−/−)*^ fish. Finally, the ubiquitin‐mediated protein degradation pathway was significantly disrupted in the *ngly1* ^(−/−)^ zebrafish brain, accompanied by marked amyloid aggregation. These findings provide compelling evidence of global dysfunction in key aspects of proteostasis, encompassing folding and degradation, further underscoring the profound impairment of cellular protein homeostasis in this model of NGLY1 deficiency (Figure 5). These findings support previous reports showing that zebrafish are highly amenable for modeling human diseases. Moreover, this study demonstrated that the zebrafish is a powerful tool for studying NGLY1 deficiency, since the mutant fish featured a unique combination of clinical hallmarks of the disease, including global developmental delay, movement disorders and eyes abnormalities [24]. Continued investigation of the zebrafish model of NGLY1 deficiency may resolve the molecular mechanisms behind this rare systemic disorder and may serve as a platform for experimental treatments for this devastating disease in humans. Moreover, it also may serve as a model for common neurodegenerative diseases that include motor cognitive decline and movement impediment.

## Author Contributions

Aviv Mesika designed and performed the experiments, designed the microscopy analyses and behavioral analyses of the fish, and wrote the first draft. Golan Nadav participated in the design of experiments, executed experiments, prepared figures, and participated in writing the original draft. Sapir Ben‐David participated in AQP1 experiments. Limor Kalfon supervised molecular studies, tissue cultures, and expression analyses. Chen Shochat participated in micro‐CT experiments. Rana Nasra participated in the histology experiments. Alejandro Livoff supervised the pathology study. David Karasik supervised fish experiments and wrote and edited the manuscript. Tzipora C. Falik‐Zaccai conceived and designed the project, supervised the experiments and interpretation of the data, wrote the paper, and approved the final version.

## Ethics Statement

The guardians of the affected individuals provided signed informed consent for the children to participate in this study. The Israeli Supreme Helsinki committee approved the study; GMC 03‐04‐2006. All animal research followed a protocol approved by BIU IACUC (# 023_b15280_80).

## Consent

The authors have nothing to report. All institutional and national guidelines for the care and use of laboratory animals were followed.

## Conflicts of Interest

The authors declare no conflicts of interest.

## Supporting information


**Figure S1.** *ngly1* ^*(−/−)*^ adult fish show no significant injury in the liver compared to *ngly1* ^*(+/+)*^. (A) *ngly1* ^*(+/+)*^ and (B) *ngly1* ^*(−/−)*^ liver section histology staining (H&E), (A, B: X20). (C) Adult (12 month old) fish and (D) larval stage measurement of serum ALT and AST activity; T‐test (*N* = 5 in each group of adults, *N* = 120 in each group of the larval stage (6 *dpf*), *p* > 0.1).
**Figure S2:** Quantification of osteogenesis and ossification in larval fish (7 *dpf*). (A) Schematic representation of viscerocranium skeleton 7 *dpf*: teeth (T), ceratobranchial (cb), hyosymplectic (hs), palatoquadrate (pq), ceratohyal (ch), and Meckel’s cartilage (m). (B) Representative image of *ngly1* ^*(+/+)*^ 7 *dpf* zebrafish bone and cartilage stained with Alizarin red and Alcian blue (*N* = 14 in each group). (C) *ngly1* ^*(−/−)*^ 7 *dpf* zebrafish bone and cartilage stained with Alizarin red and Alcian blue. (D) Notochord staining intensity was analyzed in *ngly1* ^*(−/−)*^ 7 *dpf* zebrafish and *ngly1* ^*(+/+)*^; ossification unit = quantification of notochord pixel intensity. *N* = 14 in each group. Scale bars:1 mm.

## Data Availability

The data underlying this work are available in the article and in its online Supporting Information.
