# Supplementary material for: Impaired Proteostasis is Linked to Neurological Pathology in a Zebrafish NGLY1 Deficiency Model
Source: J Inherit Metab Dis. 2025 Jun 5;48(4):e70050. doi: 10.1002/jimd.70050 (PMC12138962; doi:10.1002/jimd.70050)
Supplement: Supplementary file 1 — Figure S1. ngly1 (−/−) adult fish show no significant injury in the liver compared to ngly1 (+/+) . (A) ngly1 (+/+) and (B) ngly1 (−/−) liver section histology staining (H&E), (A, B: X20). (C) Adult (12 month old) fish and (D) larval stage measurement of serum ALT and AST activity; T‐test (N = 5 in each group of adults, N = 120 in each group of the larval stage (6 dpf), p > 0.1). Figure S2: Quantification of osteogenesis and ossification in larval fish (7 dpf). (A) Schematic representation of viscerocranium skeleton 7 dpf: teeth (T), ceratobranchial (cb), hyosymplectic (hs), palatoquadrate (pq), ceratohyal (ch), and Meckel’s cartilage (m). (B) Representative image of ngly1 (+/+) 7 dpf zebrafish bone and cartilage stained with Alizarin red and Alcian blue (N = 14 in each group). (C) ngly1 (−/−) 7 dpf zebrafish bone and cartilage stained with Alizarin red and Alcian blue. (D) Notochord staining intensity was analyzed in ngly1 (−/−) 7 dpf zebrafish and ngly1 (+/+) ; ossification unit = quantification of notochord pixel intensity. N = 14 in each group. Scale bars:1 mm. [file JIMD-48-0-s001.docx]

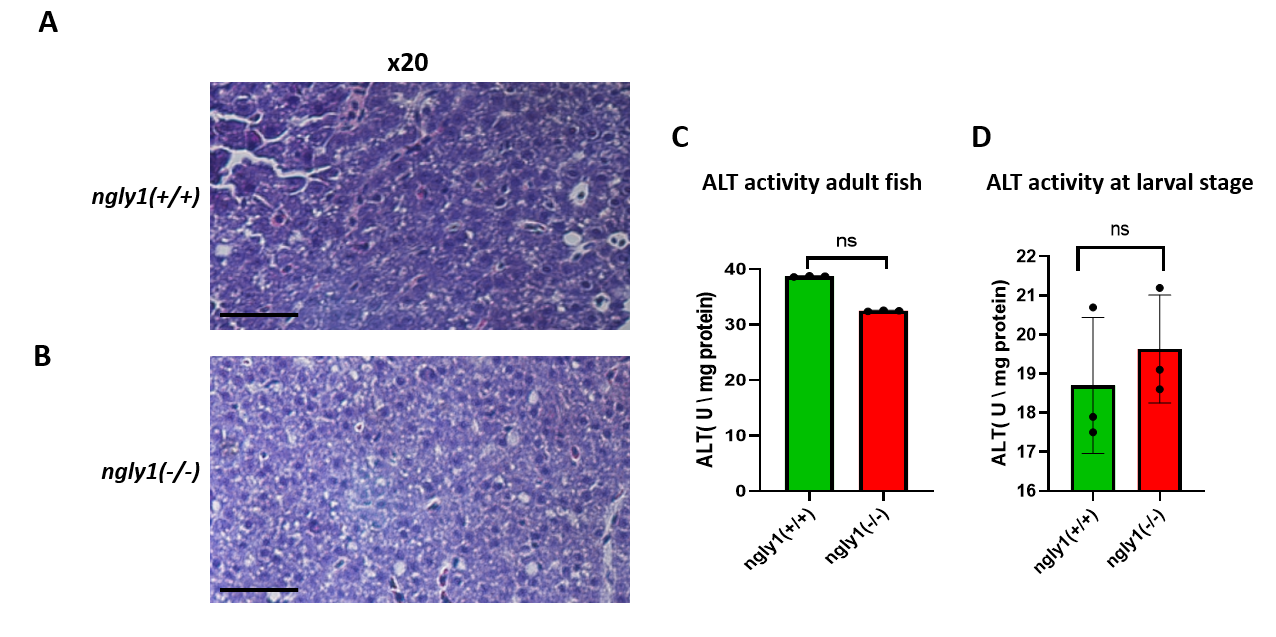
**Supplementary Figures**

**Figure S1: *ngly1^(-/-)^* adult fish show no significant injury in the liver compared to *ngly1^(+/+)^***. **(A)** *ngly1^(+/+)^* and **(B)** *ngly1^(-/-)^* liver section histology staining (H&E), (A-B X20). **(C)** adult (12 mo old) fish and **(D)** larval stage measurement of serum ALT and AST activity; T-test (N=5 in each group of adults, N=120 in each group of larval stage (6*dpf*), p>0.1).


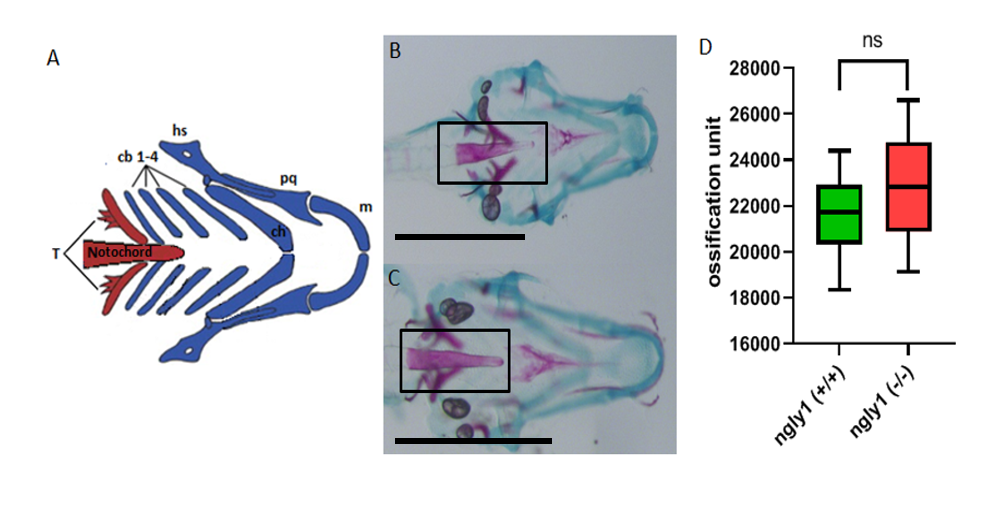


**Figure S2: Quantification of osteogenesis and ossification in larval fish (*7dpf*). (A)** Schematic representation of viscerocranial skeleton 7*dpf*: teeth (T), ceratobranchial (cb), hyosymplectic (hs), palatoquadrate (pq), ceratohyal (ch), Meckel’s cartilage (m). **(B)** Representative image of *ngly1^(+/+)^* 7*dpf* zebrafish bone and cartilage stained with Alizarin red and Alcian blue (N=14 in each group). **(C)** *ngly1^(-/-)^* 7*dpf* Zebrafish bone and cartilage stained with Alizarin red and Alcian blue. **(D)** Notochord staining intensity was analyzed in *ngly1^(-/-)^* 7*dpf* zebrafish and *ngly1^(+/+)^*; ossification unit =quantification of notochord pixel intensity. N=14 in each group. Scale bars- 1mm.
